# The Centrosomal Protein Pericentrin Identified at the Basal Body Complex of the Connecting Cilium in Mouse Photoreceptors

**DOI:** 10.1371/journal.pone.0026496

**Published:** 2011-10-21

**Authors:** Johanna Mühlhans, Johann Helmut Brandstätter, Andreas Gießl

**Affiliations:** Department of Biology, Animal Physiology, University of Erlangen-Nuremberg, Erlangen, Germany; University of Oldenburg, Germany

## Abstract

**Background:**

Pericentrin (Pcnt), a conserved protein of the pericentriolar material, serves as a multifunctional scaffold for numerous proteins and plays an important role in microtubule organization. Recent studies indicate that Pcnt mutations are associated with a range of diseases including primordial dwarfism and ciliopathies. To date, three Pcnt splice variants from orthologous genes in mice and humans are known.

**Principal Findings:**

We generated a specific Pcnt antiserum detecting all known Pcnt splice variants and examined the cellular and subcellular distribution of Pcnt in ciliated tissues of the mouse, the olfactory epithelium and the retina. For the first time, we identified Pcnt and its centrosomal interaction partners at the basal body complex of mouse retinal photoreceptors. Photoreceptors are morphologically and functionally subdivided into the light sensitive outer segment and the inner segment comprising the metabolic function of the cell. The two compartments are linked via a modified, specialized, non-motile cilium, the connecting cilium. Here, Pcnt colocalized with the whole protein machinery responsible for transport processes between the two compartments. Surprisingly, photoreceptors expressed a small Pcnt splice transcript – most likely a modified variant of Pcnt S – which was not present in receptor neurons of the olfactory epithelium.

**Conclusions:**

Our findings suggest distinct functional roles of several Pcnt variants in different ciliated tissues and sensory neurons, like the olfactory epithelium and the retina of the mouse. The individual patchwork of different Pcnt splice transcripts seems to reflect the complexity of Pcnt function, an assumption corroborated by the heterogeneous clinical manifestations associated with mutations in the Pcnt gene.

## Introduction

Pericentrin (Pcnt), also known as kendrin, is an integral component of the centrosome. It was first identified as a large coiled-coil protein that serves as a multifunctional scaffold for numerous proteins and protein complexes [1], [2], [3]. To date, up to three Pcnt splice variants from orthologous genes in mice and humans – Pcnt B (360), Pcnt A and Pcnt S (250) – are known [4], [5], [6], [7]. Structurally, Pcnt is characterized by coiled-coil domains throughout most of the protein and by a pericentrosomal matrix targeting motif, called the PACT [pericentrin-AKAP450 (AKAP9) centrosomal targeting] domain [1], [4], [8], [9]. Through its many interactions, Pcnt contributes to a diversity of fundamental cellular processes (for a review see [10]). At centrosomes, for example, Pcnt is suggested to be involved in microtubule nucleation and anchoring as well as in the regulation of multiple cell cycle transitions [3].

Centrosomes are the precursors of primary cilia, i.e. non-motile sensory organelles found on most vertebrate cells. As primary cilia are involved in several human diseases, the role of centrosomal proteins in ciliary function has obtained increasing attention in recent years [11]. The involvement of Pcnt in ciliogenesis has been demonstrated in cultured human epithelial cells, where Pcnt depletion by RNA interference inhibits primary cilia assembly [12]. Moreover, Pcnt has been shown to be localized at the base of primary cilia in multiple embryonic tissues [13]. In Drosophila, the Pcnt orthologue D-PLP (Drosophila pericentrin-like protein) is essential for normal ciliogenesis. Depletion of D-PLP causes malformed sensory cilia in the fly's mechanosensory and chemosensory neurons [14]. In the sensory cells of the mouse olfactory epithelium, a Pcnt splice variant is required for normal olfactory cilia assembly, but not for the correct assembly of cilia in other sensory neurons, e.g. the photoreceptors in the retina [15]. In humans, recent genetic studies indicate that Pcnt mutations are associated with a rare autosomal recessive genetic disorder – Majewski/microcephalic osteodysplastic primordial dwarfism type II (MOPD II) [16], [17]. The disease is characterized by primordial dwarfism and microcephaly, often accompanied by mental retardation [6].

The aim of the present study was to characterize the expression patterns of the known and putative new Pcnt variants in mouse tissues with a particular focus on the retina and its sensory neurons, the photoreceptors. In the vertebrate retina, the light sensitive outer segment of the photoreceptor is linked via a small intracellular bridge, the connecting cilium, with the inner segment. The inner segment contains the typical energy producing and protein nthesizing components of an eukaryotic cell [18]. The connecting cilium is a modified and specialized, non-motile primary cilium [19]. We found Pcnt and several Pcnt interaction partners, which are known from the centrosome and from primary cilia, at the basal body complex (BBC) of the photoreceptor's connecting cilium. Here Pcnt is colocalized with its interaction partners and the protein machinery that regulates the ciliary transport of proteins from the inner to the outer segment [19], [20], [21], [22], [23], [24]. The presence of Pcnt at the BBC of the connecting cilium and its interaction with transport molecules at primary cilia like the intraflagellar transport proteins (IFTs) [12] suggest a role for Pcnt in photoreceptor ciliary transport functions. In summary, our study provides evidence that Pcnt splice variants are differentially expressed in different tissues and sensory neurons, where Pcnt may fulfill diverse functions as a component of the ciliary regulation and transport network.

## Results

It is known that mutations in the PCNT gene in humans cause autosomal recessive genetic disorders accompanied by primordial dwarfism [6], [16], [17], but the function of Pcnt is not yet understood. Pcnt interacts with several proteins at the centrosome, e.g. the pericentriolar material 1 (PCM1) and the centrosomal and Golgi N-kinase anchoring protein (CG-NAP) [2], [25], [26]. Together these proteins play a role in centrosomal organization and cell cycle progression [3], . Pcnt is also present at the base of primary cilia, where it recruits numerous proteins and is essential for primary cilia assembly in cell culture [12].

### Generation and characterization of an antiserum against Pericentrin

**Figure 1:**
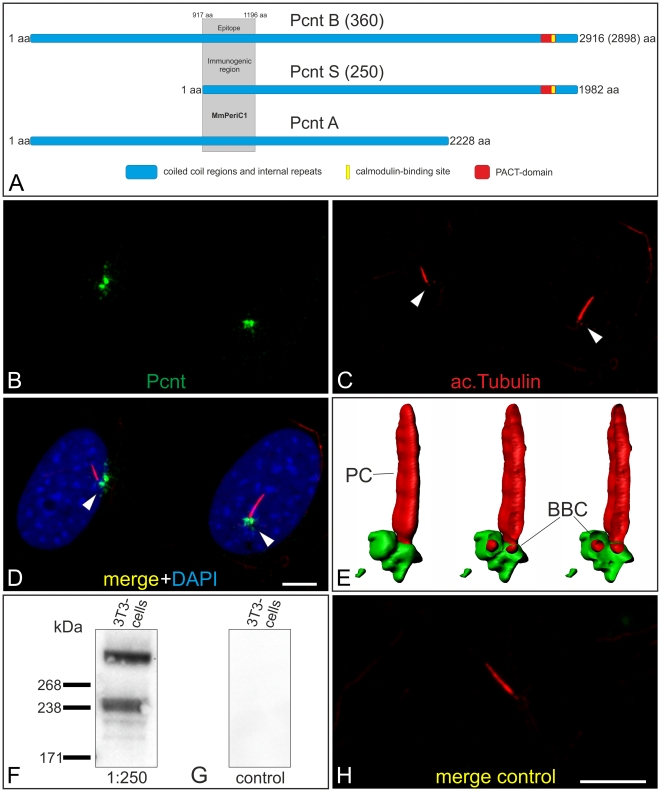
Characterization of the Pericentrin antiserum MmPeriC1. (A) Scheme of the known and published Pcnt splice variants: Pcnt B (360, accession number (AN): NP_032813 or BAF36559), Pcnt A (AN: partial, AAO24322.1) and Pcnt S (250, BAF36560). The 280 amino-acid epitope against which the MmPeriC1 antiserum was generated is indicated in grey. (B-D) Triple labeling of Pcnt (B, green), ac. tubulin (C, red), and DAPI (D merge, blue) in NIH 3T3 mouse fibroblasts. Pcnt colocalizes partially with ac. tubulin at the BBC (arrowheads) of primary cilia (PC). (E) High resolution 3D reconstruction demonstrates that Pcnt (green) ensheaths the BBC of the PC (red, ac. tubulin). (F, G) Western blot analysis using the MmPeriC1 antiserum. (F) The antiserum detects a 360 kDa and a 240–250 kDa protein band. (G) Preincubation of the MmPeriC1 antiserum with the antigen (recombinant fusion protein) blocks the immunodetection of the two bands. (H) Preadsorption of the MmPeriC1 antiserum with the antigen results in the absence of Pcnt staining at the BBC at primary cilia labeled with ac. tubulin. Scale bar: 5 µm (D, H).

To study the localization of Pcnt in the olfactory epithelium and the retina of the mouse, we first generated and affinity purified a specific polyclonal antiserum against a recombinant expressed part of murine Pcnt (Fig. 1A, Fig. S3A). The Pcnt antiserum, MmPeriC1, should detect all to date known Pcnt splice variants because of the immunogenic region against which the MmPeriC1 antiserum is directed (Fig. 1A). To validate the specificity of the MmPeriC1 antiserum, we examined the localization of Pcnt in cultured NIH 3T3 mouse fibroblasts, a cell system in which the formation of primary cilia can be induced by starvation. Famished NIH 3T3 mouse fibroblasts stop dividing, they get arrested in the G_0_ phase and primary cilia assembly occurs. Triple labeling and high resolution immunofluorescence imaging of induced primary cilia in resting NIH 3T3 mouse fibroblasts demonstrated that Pcnt partially overlapped with acetylated tubulin (ac. tubulin), a marker for the ciliary axoneme and the BBC at the base of primary cilia (Fig. 1B-E). Double labeling experiments for Pcnt and centrin3 (Cen3), which is present at the transition zone of the ciliary axoneme and the BBC [19], gave comparable results (data not shown). The BBC consists of the basal body (BB) and its centriole where the pericentriolar material (PCM) is localized. High resolution 3D reconstructions of the cilia and their BBCs demonstrated that Pcnt surrounds the BBC of primary cilia (Fig. 1E). In Western blot analyses of extracts of NIH 3T3 mouse fibroblasts, the MmPeriC1 antiserum recognized two protein bands at approximately 360 and 240–250 kDa corresponding to the predicted size of the holo protein Pcnt B (360) and of the Pcnt splice variants A and/or S (250) [1] (Fig. 1A, F). Preadsorption of the MmPeriC1 antiserum with an excess of the antigen used for immunization completely blocked the immunodetection of the two protein bands in the Western blot (Fig. 1G) and resulted in the absence of Pcnt staining at the BBC of primary cilia of NIH 3T3 mouse fibroblasts (Fig. 1H). Pcnt expression was additionally examined using a polyclonal Pcnt pAb (Covance) which was raised against an epitope common to all three isoforms. The comparison of Western blot analyses with the MmPeriC1 and the Covance Pcnt antiserum using various tissue extracts showed for both antisera the same Pcnt bands (Fig. S3) [7], [15]. These results demonstrate the specificity of the affinity purified MmPeriC1 antiserum. In our study we did not use the anti-Pcnt monoclonal antibody (Pcnt mAb; BD Biosciences), because Endoh-Yamagami and co-workers clearly showed that this antibody does not detect all Pcnt splice variants [7].

**Figure 2:**
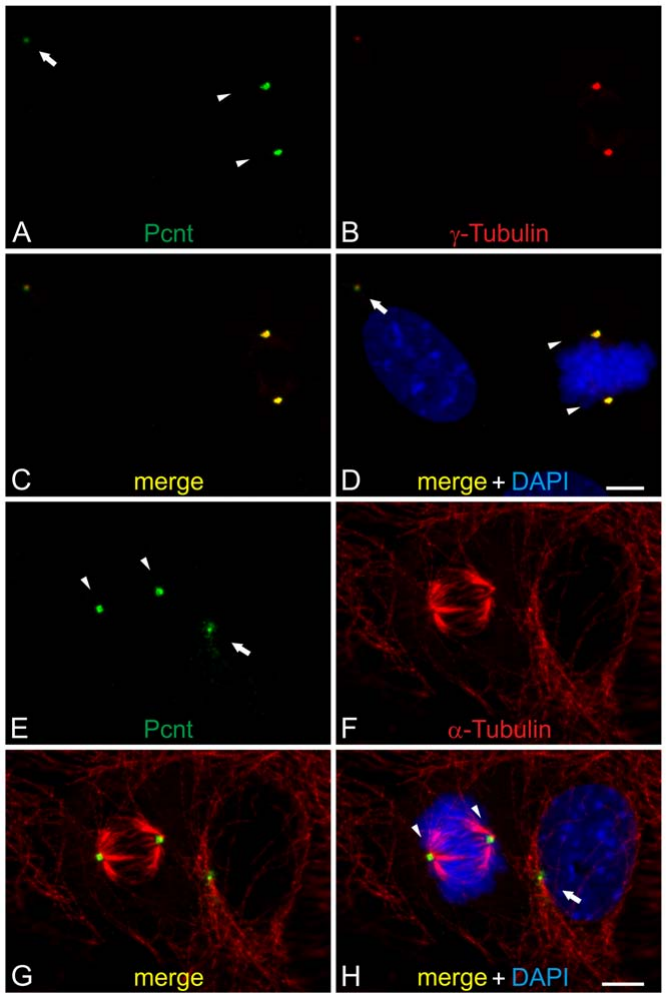
Localization of Pericentrin during the cell cycle in NIH 3T3 mouse fibroblasts. (A–D) Triple staining of endogenous Pcnt (A, green), γ-tubulin (B, red) as a centrosomal marker, and DAPI (D, blue) as a nuclear marker in NIH 3T3 mouse fibroblasts. (C–D) The merge of the stainings shows the localization of Pcnt at the centrosomes in dividing (arrowheads) and in non-dividing cells (arrow). (E–H) NIH 3T3 mouse fibroblast cells stained for endogenous Pcnt (E, green), α-tubulin (F, red) as a marker for microtubules, and DAPI (H, blue). (G–H) In the merge pictures Pcnt is localized at the centrosomes. Scale bars: 5 µm (H, D).

Additional immunocytochemical stainings of active NIH 3T3 mouse fibroblasts with the MmPeriC1 antiserum revealed that Pcnt overlapped with γ-tubulin, a protein associated with the mother and daughter centriole of the centrosome in all phases of the cell cycle (Fig. 2A–D). Centrosomal structures also colocalize with α-tubulin, a marker for microtubules (Fig. 2E–H). These results indicate the presence of Pcnt at centrosomal or BBC structures in every stage of the cell cycle, regardless of whether the cell is dividing or resting, and support the assumption that Pcnt may also play a role in resting, and thus often ciliated cells.

### Localization of Pericentrin at cilia of the olfactory epithelium in the mouse

**Figure 3:**
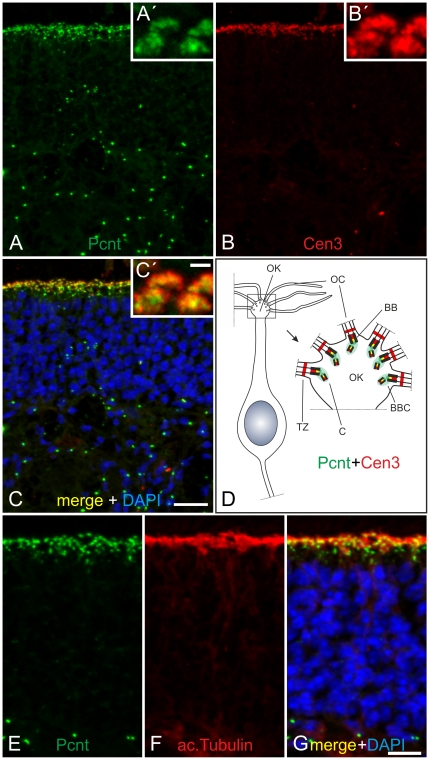
Localization of Pericentrin at mouse olfactory cilia. (A–C) Micrographs of a cryostat section through an adult mouse olfactory epithelium double labeled with the MmPeriC1 antiserum against Pcnt (A and A', green) and an antibody against Cen3 (B and B', red), as a marker for the ciliary apparatus. (A) Pcnt is localized in the ciliary region of the olfactory receptor neurons and at the centrosomes of the other cells in the mouse olfactory epithelium. (A') The higher magnification shows a punctate staining for Pcnt inside the olfactory knobs of the receptor neurons. (B) Cen3, like Pcnt, is localized in the ciliary region of the olfactory receptor neurons and at the centrosomes of the other cells in the mouse olfactory epithelium. (B') The higher magnification shows that Cen3 is preferentially localized at the ciliary surface of the olfactory knobs of the receptor neurons. (C) Merge of the stainings for Pcnt (green) and Cen3 (red) combined with a DAPI nuclear staining (blue). (C') The higher magnification of the merged signals shows a partial colocalization of Pcnt and Cen3 at the ciliary apparatus of the olfactory knobs of the receptor neurons. (D) Schematic drawing of an olfactory receptor neuron and a higher-power view of its olfactory knob with the localization of Pcnt (green) at the basal body complexes (BBCs) and of Cen3 (red) at the transition zones (TZs) of the BBCs of the olfactory cilia (OC). (E–G) Micrographs of a section through an adult mouse olfactory epithelium double labeled for Pcnt (E, green) and ac. tubulin (F, red) as a marker for the axoneme of olfactory cilia. (E) Pcnt is localized in the ciliary region of the olfactory receptor neurons and at the centrosomes of other cells in the mouse olfactory epithelium. (F) Ac. tubulin, like Pcnt, is localized in the ciliary region of the olfactory receptor neurons. (G) The merge of the stainings combined with a DAPI nuclear stain (blue) shows a partial colocalization of Pcnt and ac. tubulin in the ciliary region of the olfactory receptor neurons. C: centriole; BB: basal body. Scale bars: 20 µm (C G), 5 µm (C').

D-PLP is the orthologue protein of Pcnt in Drosophila, and *D-PLP*-mutant flies were shown to display malformed sensory cilia in the mechanosensory and chemosensory neurons [14], [29]. Ciliated sensory neurons are also present in mammals, e.g. the receptor neurons of the olfactory epithelium, which are specialized in odorant detection. In 2009 Miyoshi and co-workers showed that splice variants of Pcnt are essential for the assembly of the chemosensory cilia in the olfactory receptor neurons. To verify the localization of Pcnt in the olfactory receptor neurons of wild type mice, we stained cryostat sections of the olfactory epithelium with the MmPeriC1 antiserum. To show the exact localization of Pcnt at the olfactory cilium of the receptor neurons, we used the Cen3 antibody as a marker of the transition zone of the ciliary axoneme and the BBC [19], [21]. Pcnt was partially colocalized with Cen3, which is consistent with the position of the BBCs in the dendritic knob of the olfactory receptor neurons in the wild type mouse (Fig. 3A–D). The localization of Pcnt at the BBCs below the ciliary layer is best seen in the high power views shown in the insets in Figure 3 (Fig. 3A'–C'). Additional double labeling experiments using ac. tubulin to mark the complete ciliary axoneme confirmed the localization of Pcnt at the olfactory cilium (Fig. 3E–G). In addition to Pcnt labeling in the receptor neurons, Pcnt was present at the centrosomes of the other cell types in the mouse olfactory epithelium (Fig. 3).

### Localization of Pericentrin at the ciliary apparatus of photoreceptors in the mouse retina

**Figure 4:**
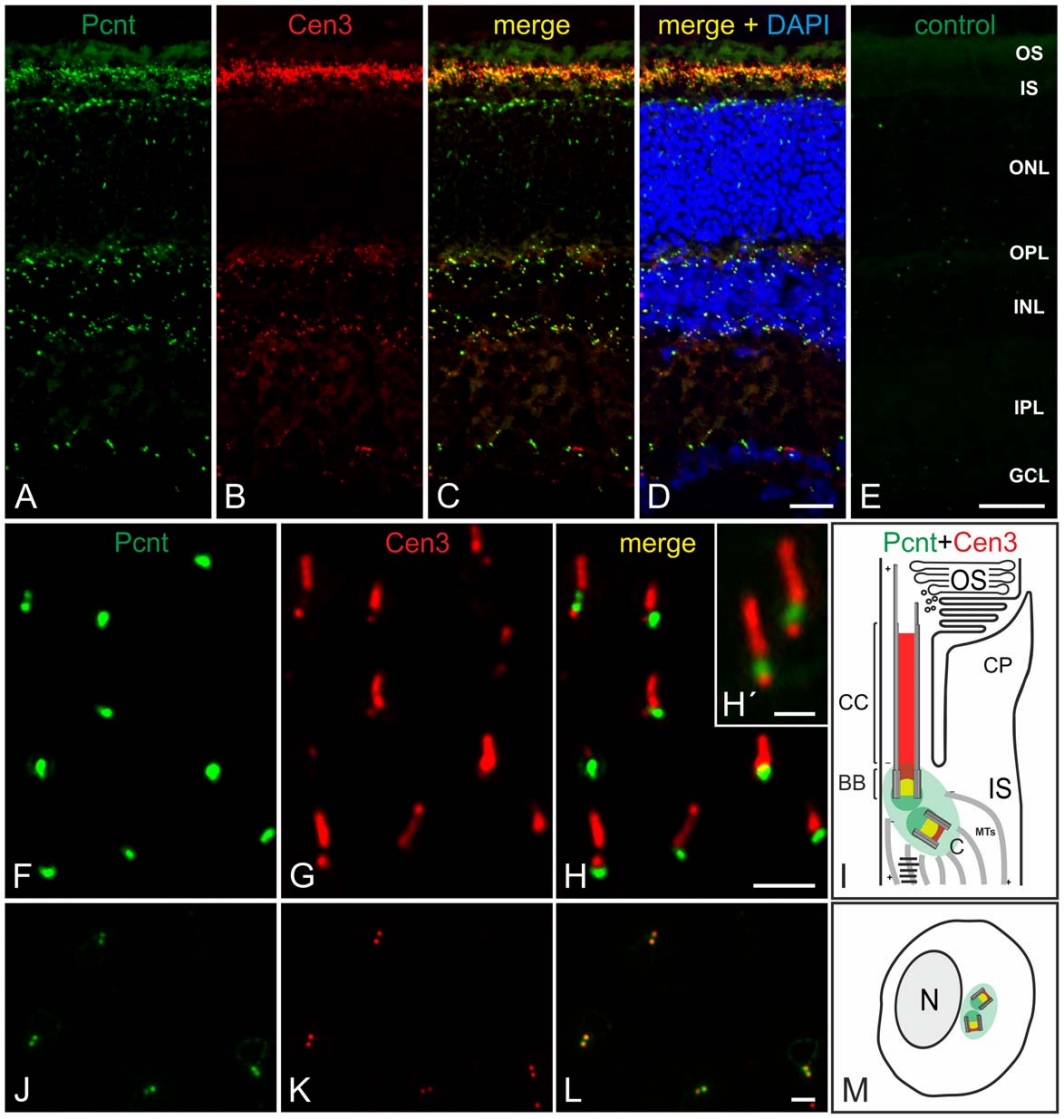
Localization of Pericentrin at connecting cilia and at centrosomes in the mouse retina. (A–D) Micrographs of a vertical cryostat section through an adult mouse retina double labeled for Pcnt (A, green) and Cen3 (B, red). (C, D) As seen in the merge of the stainings with the additional labeling of the cell nuclei with DAPI, Pcnt and Cen3 colocalize at the ciliary region of the photoreceptors and at the centrosomes of the other retinal cells. (E) Preadsorption control of the MmPeriC1 antiserum with its antigenic peptide results in the absence of Pcnt labeling. (F–M) Subcellular localization of Pcnt (F, J, green) and Cen3 (G, K, red) at the BBC of the photoreceptor connecting cilia (F–H) and at the centrosomes of non-photoreceptor cells in the INL (J–L). (H') Higher power view showing the partial colocalization of Pcnt and Cen3 at the BBC. (L) In non-photoreceptor cells, Pcnt and Cen3 colocalize at the centrioles of the centrosomes. (I, M) Schemes summarizing the subcellular localization of Pcnt and Cen3 at the connecting cilium of photoreceptors (I) and at the centrosomes of non-photoreceptor cells (M). OS: outer segment; IS: inner segment; ONL: outer nuclear layer; OPL: outer plexiform layer; INL: inner nuclear layer; IPL: inner plexiform layer; GCL: ganglion cell layer; BB: basal body; C: centriole; CC: connecting cilium; CP: calycal process; MTs: microtubules; N: nucleus. Scale bars: 20 µm (D, F), 2 µm (I), 1 µm (I'), 2 µm (M).

To determine the distribution of Pcnt in the mouse retina, cryostat sections through whole unfixed eyes were analyzed with immunofluorescence microscopy using the MmPeriC1 antiserum. Punctate staining for Pcnt was present in the region between the inner (IS) and the outer segments (OS) of the photoreceptors, in the outer nuclear layer (ONL), the inner nuclear layer (INL), and the ganglion cell layer (GCL) (Fig. 4A–D). Double labeling with the Cen3 antibody, which stained the whole photoreceptor connecting cilium corresponding to the transition zone of a prototypic cilium [19], showed a colocalization of Pcnt and Cen3 at the ciliary region of the photoreceptors and at the centrosomes of the other cells in the retina (Fig. 4C, D). High magnification imaging further revealed a partial colocalization of Pcnt and Cen3 at the BBC of the photoreceptor's connecting cilium (Fig. 4F–I, Fig. S1A–D), and, like in other primary cilia (Fig. 1E), Pcnt is present at the PCM, which ensheaths the BBC (Fig. 4I, Fig. S1). Additional high resolution 3D reconstructions of the connecting cilium stained with Pcnt and Cen3 (Fig. S1A–D) and with Pcnt and ac. tubulin (Fig. S1F–I) demonstrate that Pcnt surrounds the BBC of the connecting cilium in photoreceptors (Fig. S1). These results assign Pcnt as a component of the BBC and the PCM of the connecting cilium in photoreceptors, the only intracellular bridge between the metabolic inner and the light sensitive outer segment of the photoreceptor. In the cells of the INL and the GCL, Pcnt localized to the mother and daughter centrioles and to the PCM of the centrosomes. Here Pcnt colocalized with Cen3 (Fig. 4J–M, Fig. S1E) and other centrosomal markers (data not shown).

**Figure 5:**
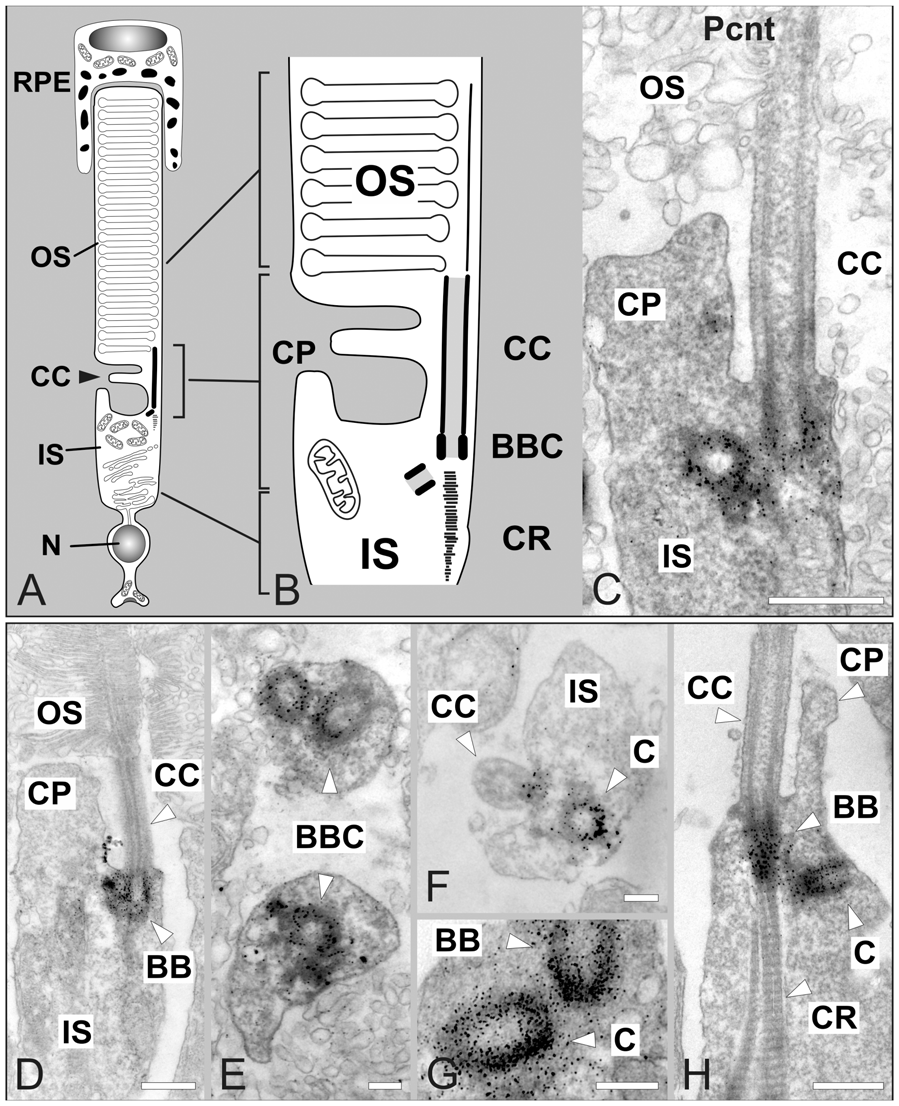
Immunoelectron microscopic localization of Pericentrin in mouse photoreceptors. (A) Scheme of a mouse rod photoreceptor with the tip of the photoreceptor’s light-sensitive outer segment (OS) enclosed by the retinal pigment epithelium (RPE). (B) Scheme of the ciliary apparatus. The photosensitive OS is linked by the connecting cilium (CC) to the metabolic inner segment (IS), which includes the basal body complex (BBC) in its apical region. (C–H) Electron micrographs showing the ultrastructural localization of Pcnt in the photoreceptor ciliary region. Longitudinal (C, D, G, H) and cross sections (E, F) through the ciliary region show the presence of Pcnt at the basal bodies (BB) (C, D, G) but not in other parts of the CC (F, H). In the apical region of the IS, Pcnt is located at the centriole (C) (F, G, H) at the BBC (E) but not in the cytoplasm of the calycal process (CP) (C, D, H). N: nucleus; CR: ciliary rootlet. Scale bars: 0.5 µm (B, C, D, H), 0.2 µm (E, F, G).

To confirm the Pcnt localization at the BBC of the photoreceptor connecting cilium, we performed an immunoelectron microscopical analysis using the MmPeriC1 antiserum (Fig. 5). Figure 5A shows a schematic illustration of a rod photoreceptor and an enlarged view of the ciliary region between the inner and the outer segment of the photoreceptor is shown in Figure 5B. Employing the method of preembedding immunoelectron microscopy [24], we successfully localized Pcnt at the region of the BBC (Fig. 5C–H). Pcnt was present at the surface of the microtubules of the BB and its centriole (Fig. 5C, D, G, H). Furthermore Pcnt staining surrounded the BBC, where the PCM is located (Fig. 5E, G). No labeling for Pcnt was found inside the connecting cilium (Fig. 5F). Our immunoelectron microscopical examination also confirmed the localization of Pcnt at the centrioles of the centrosomes of non-photoreceptor cells in the retina (data not shown).

### Localization of centrosomal and ciliary interaction partners of Pericentrin at the connecting cilium

Pcnt is a multifunctional scaffold for the anchoring of a number of centrosomal proteins, and it is involved in many essentially functions of the cell [10]. We therefore asked the question, whether known centrosomal and ciliary interaction partners of Pcnt can also be found at the connecting cilium of the photoreceptors. In order to make sure that the analyzed interaction partners of Pcnt are present in the mouse retina, we first performed a RT-PCR expression analysis (data not shown). In the following immunocytochemical analyses, we could not perform direct double labeling experiments because the available antibodies detecting all splice variants of Pcnt (including MmPeriC1) [7] and its known interaction partners were all from the same species. To circumvent this problem, we used Cen3 as a marker for the localization of Pcnt because Cen3 and Pcnt colocalize in the ciliary region of the photoreceptors and at the centrosomes of the other retinal cells (Fig. 4).

**Figure 6:**
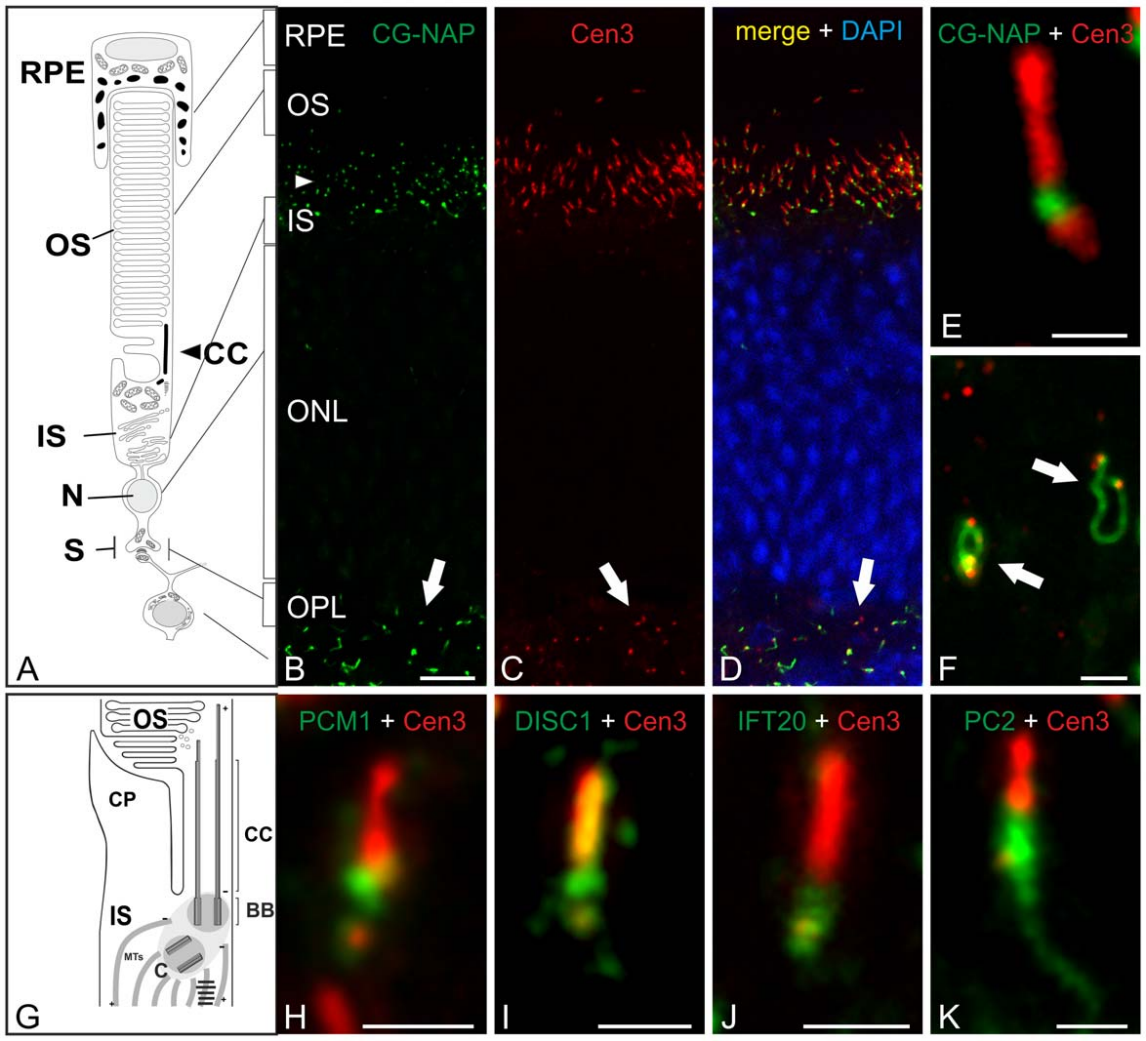
Colocalization of various Pericentrin interaction partners at the basal body complex of mouse photoreceptors. (A) Scheme of a vertebrate rod photoreceptor. (B–F) Micrographs of a vertical cryostat section through an adult mouse retina double labeled for CG-NAP (B, green) and Cen3 (C, red) as a marker for the ciliary apparatus (the connecting cilium (CC) and the basal body complex (BBC)) and the centrosomes. (B) CG-NAP and (C) Cen3 are localized in the ciliary region of the photoreceptors and at the centrosomes of the other retinal cells. (D) The merge of the stainings with the additional DAPI nuclear staining (blue) shows the partial colocalization of CG-NAP and Cen3 in the ciliary region of the photoreceptors and the centrosomes of the other retinal cells. (E–F) High power views showing the localization of CG-NAP at the BBC of the connecting cilium (E) and at the centrosomes of the other retinal cells (F). (G) Scheme of the ciliary apparatus. (H–K) High power views showing the localization of the Pcnt interaction partners PCM1 (H, green), DISC1 (I, green), IFT20 (J, green), and PC2 (K, green) at the BBC of the connecting cilium of the photoreceptor. Cen3 (red) marks the whole cilium with the ciliary apparatus. RPE: retinal pigment epithel; OS: outer segment; IS: inner segment; ONL: outer nuclear layer; OPL: outer plexiform layer; BB: basal body; CC: connecting cilium; C: centriole; CP: calycal process; MTs: microtubules; N: nucleus; S: synapse. Scale bars: 10 µm (B), 1 µm (E, F, J, K, H, I).

A centrosomal interaction partner of Pcnt is CG-NAP, a giant coiled-coil protein, which is found at the centrosome throughout the cell cycle and at the Golgi apparatus during interphase [2]. The double labeling experiments with antibodies against CG-NAP and Cen3 showed a partial colocalization of the two proteins at the ciliary region of the photoreceptors and at the centrosomes of the other cells in the retina (Fig. 6A–F). As seen in the higher power view of the photoreceptor ciliary region (Fig. 6E), CG-NAP is present at the BBC of the connecting cilium, the site where Pcnt is also found (Fig. 4, Fig. 5, Fig. S1). At the other retinal cells, CG-NAP is found at the centrosomes and at parts of the Golgi apparatus (Fig. 6F) [2]. Other known centrosomal interaction partners of Pcnt, which we detected at the BBC of the photoreceptor's connecting cilium, were the proteins PCM1 [25], [26], [30] (Fig. 6H) and DISC1 (disrupted-in-schizophrenia 1) [31], [32] (Fig. 6I). DISC1 is also known to be involved in the regulation of the formation and/or the maintenance of primary cilia and in establishing the integration of dopamine receptors to the ciliary surface [33], [34].

For known ciliary interaction partners of Pcnt, we focused on IFT20 and the Ca^2+^ release channel PC2 (polycystin-2) [12]. Both proteins form a complex with Pcnt, and the depletion of Pcnt causes a disruption of centriole association and primary cilia formation [12]. In agreement with the results of a study by Sedmak and Wolfrum (2010), we detected IFT20 at the BBC of the connecting cilium of photoreceptors (Fig. 6J), where PC2 is also localized (Fig. 6K). The presence of centrosomal and ciliary interaction partners of Pcnt at the connecting cilium, the bridge between the inner and outer segment of the photoreceptor, suggests a role for Pcnt in ciliary regulation and transport function.

### Expression of Pericentrin splice variants in ciliated tissues of the mouse

**Figure 7:**
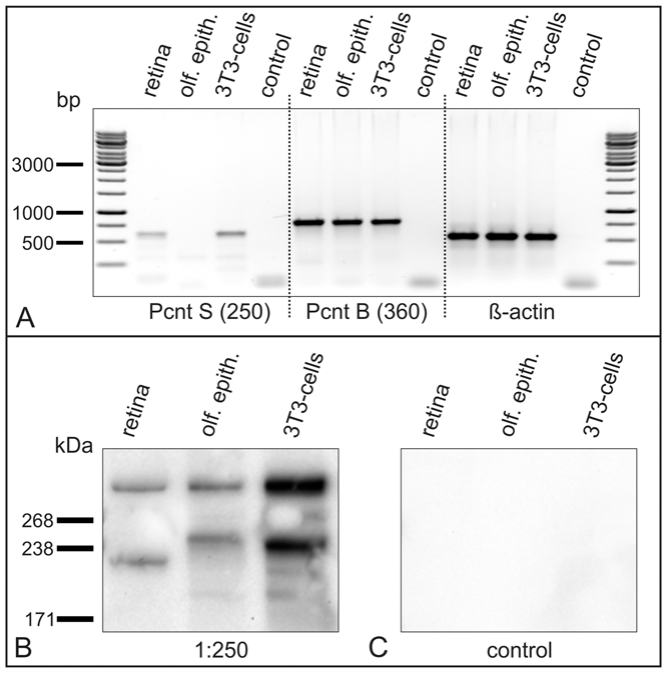
Expression of Pericentrin splice variants in different mouse tissues. (A) RT-PCR made with cDNA from retina, olfactory epithelium (olf. epith.) and NIH 3T3 mouse fibroblasts using specific primer sets for Pcnt S, Pcnt B and β-actin as a positive control. All three primer sets and the used reagents were tested by a negative control without cDNA (control). Pcnt S is expressed in the retina and in NIH 3T3 mouse fibroblasts, but not in the olfactory epithelium. Pcnt B is expressed in all three tested probes. The β-actin positive control demonstrates the approximate amount and the quality of the used cDNA. (B) Western blot analysis of protein extracts of retina, olfactory epithelium and NIH 3T3 mouse fibroblasts using the MmPeriC1 antiserum. A 360 kDa protein band is detected in all three samples. A second protein band with varying molecular weight – approximately 250 kDa in olfactory epithelium and NIH 3T3 mouse fibroblasts, approximately 225 kDa in retina – suggests the existence of different Pcnt variants in different tissues. (C) Preadsorption control of the MmPeric1 antiserum with the respective antigen in saturating concentrations blocks the detection of the protein bands in all three tissue samples.

The Western blot analysis of NIH 3T3 mouse fibroblasts using the MmPeriC1 antiserum suggested the expression of two Pcnt splice variants (Fig. 1F). In a next step, we therefore analyzed the retina and the olfactory epithelium for the presence of Pcnt splice variants. We performed RT-PCR analysis with cDNA from the retina, the olfactory epithelium and NIH 3T3 mouse fibroblasts using highly specific primer sets for the Pcnt splice variants Pcnt S and Pcnt B (Fig. 7A). The experiments revealed that Pcnt S is expressed in the retina and in NIH 3T3 mouse fibroblasts, but not in the olfactory epithelium. However, Pcnt B is expressed in all three tested probes (Fig. 7A). In our analysis concerning Pcnt A, we found discrepancies in the sequence compared to the published sequence (GenBank/EBI/DDBJ accession number Pcnt A partial, AAO24322.1). For this reason we were not able to create a set of primers for which we could guarantee absolute specificity for the splice variant called Pcnt A and decided to concentrate only on Pcnt S and Pcnt B on mRNA-level.

In Western blot analysis of extracts of the retina, the olfactory epithelium and NIH 3T3 mouse fibroblasts the MmPeriC1 antiserum detected two protein bands of different molecular weight (Fig. 7B). In all three samples, a protein band of approximately 360 kDa, which corresponds to the predicted size of the holo protein Pcnt B, was present. A distinct lower protein band with a molecular weight of about 250 kDa, which most likely represents the known Pcnt splice variants A and/or S, was found in the olfactory epithelium and the NIH 3T3 mouse fibroblasts (Fig. 7B). Corresponding to the results of the RT-PCR, Pcnt S does not exist in the olfactory epithelium (Fig. 7A). For this reason we conclude that here the 250 kDa protein band is most likely Pcnt A. In the NIH 3T3 mouse fibroblasts we showed on mRNA-level a clear expression of Pcnt S (Fig. 7A). The protein band with a size about 240–250 kDa could in this case represent Pcnt S or Pcnt S and Pcnt A. Surprisingly, in the retinal tissue the 2 0 kDa protein band was absent, but instead a protein band of about 225 kDa was reproducibly detected (Fig. 7B). The RT-PCR showed a clear expression of Pcnt S (Fig. 7A). For this reason we conclude that this smaller protein form of Pcnt is most likely a modified variant of Pcnt S. Anyway we cannot exclude that there might also exist a smaller, modified variant of Pcnt A in that 225 kDa band. Preadsorption of the MmPeriC1 antiserum with an excess of the antigen used for immunization completely blocked the immunodetection of all protein bands in the three samples (Fig. 7C).

### Expression of Pericentrin splice variants in mouse photoreceptors

**Figure 8:**
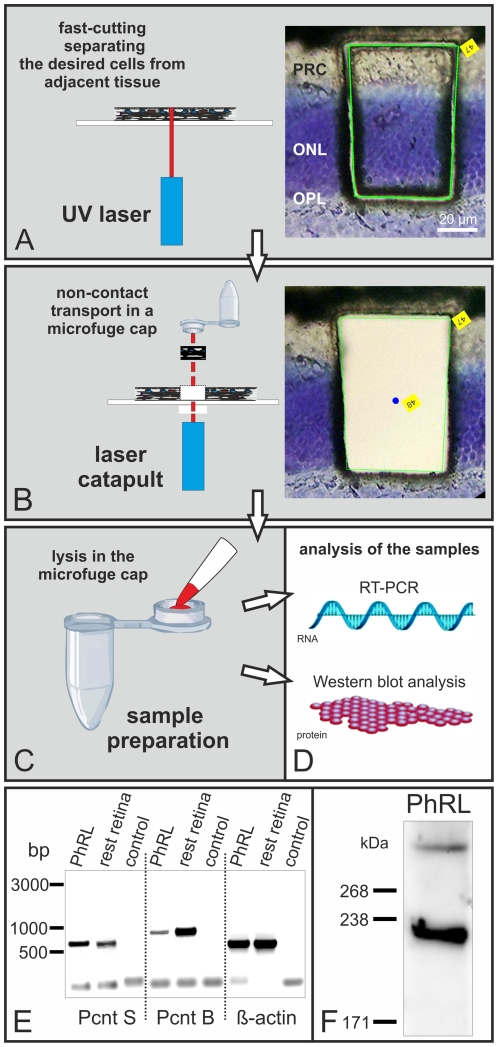
Expression of Pericentrin splice variants in separated mouse photoreceptors. (A–D) Illustration of the method of laser microdissection to separate parts of the photoreceptor cell layer or of the residual retinal layers. (E) RT-PCR made with cDNA from dissected parts of the photoreceptor cell layer (PhRL) and from dissected parts of the rest of the retina (rest retina) using specific primer sets for Pcnt S, Pcnt B and β-actin as a positive control. All three primer sets and the used reagents were tested by a negative control without cDNA (control). Pcnt S is expressed in the PhRL and in the rest of the retina, but the signal in the PhRL is slightly stronger than in the rest of the retina. Pcnt B is expressed in the PhRL and in the rest of the retina, but the signal in the PhRL is slightly weaker than in the rest of the retina. The β-actin positive control demonstrates the approximate amount and the quality of the used cDNA. (F) In Western blot analysis of extracts of dissected parts of the PhRL two specific bands with a molecular weight of approximately 360 kDa and 225 kDa were detected. The two bands represent the two Pcnt splice variants of the photoreceptors. The smaller 225 kDa Pcnt form, most likely a variant of Pcnt S, is much stronger expressed than the larger Pcnt B 360 kDa form.

To examine the expression of Pcnt in mouse photoreceptors in more detail, we first double labeled isolated mouse photoreceptors for Pcnt and Opsin, a marker for the photoreceptor outer segment (Fig. S2A–D). The two Pcnt immunoreactive puncta present at the base of the ciliary region of an isolated photoreceptor clearly identify Pcnt as a component of the BBC and the PCM of the connecting cilium (Fig. S2A–D). Knowing that two Pcnt splice variants are present in whole retinal tissue (Fig. 7A–B), we next analyzed the specific expression pattern of Pcnt in photoreceptors (Fig. 8A–F). As there are no splice variant specific Pcnt antibodies available to distinguish between the transcripts immunocytochemically, we first separated parts of the photoreceptor layer from the rest of the retina with the method of laser microdissection, followed by RT-PCR and Western blot analysis (Fig. 8A–F). The Pcnt splice variants found in whole retinal tissue (Fig. 7A–B) were also present in the photoreceptors (Fig. 8E–F). We performed RT-PCR analysis with cDNA from dissected parts of the photoreceptor cell layer and from dissected parts of the rest of the retina using highly specific primer sets for the Pcnt splice variants Pcnt S and Pcnt B. The experiments revealed that Pcnt S is slightly stronger expressed in the photoreceptor cell layer than in the rest of the retina (Fig. 8E). However, Pcnt B is slightly weaker expressed in the photoreceptor cell layer than in the rest of the retina (Fig. 8E).

In Western blot analysis of extracts of dissected parts of the photoreceptor cell layer the MmPeriC1 antiserum detected a protein band of approximately 360 kDa corresponding to the predicted size of the holo protein Pcnt B and a protein band of about 225 kDa most likely – like elucidated above - a modified variant of Pcnt S (Fig. 8F). This smaller variant of Pcnt showed a much higher protein expression level in the microdissected photoreceptors than in whole retina extract (compare Figs. 8F and 7B). These results on mRNA- and protein-level suggest a preferential expression of the smaller Pcnt variant in photoreceptors.

## Discussion

The primary cilium is located like an antenna on the surface of numerous vertebrate cell types in a single copy, where it is involved in a variety of cellular functions, e.g. in the hedgehog and wingless signaling pathways [35]. Recent studies revealed an intimate interplay between proteins of the centrosomes and the formation of primary cilia [36], [37], [38], [39], [40]. This fact reflects that in most non-dividing cells the centrioles of the centrosome migrate to the cell surface where the mother centriole forms the BB, which nucleates the nine peripheral doublet microtubules and organizes the formation of the axoneme of the primary cilium. Pcnt, first described as an integral component of the centrosome, is involved in the formation of primary cilia [1], [12]. The downregulation of Pcnt and its interaction partners by RNA interference inhibited primary cilia assembly in human epithelial cells [12], [38], [39]. The generation of a Pcnt hypomorph by the genomic integration of a trap vector decreasing the expression levels of the 360 kDa Pcnt B holo transcript disturbed the cilia assembly in receptor neurons of the olfactory epithelium in the mouse [15]. Interestingly, cilia assembly in other sensory neurons, like photoreceptors, was not affected [15].

In our study, we examined the expression and localization of Pcnt splice variants in the olfactory epithelium and the retina of the mouse with a special focus on the specialized connecting cilium of the retinal photoreceptors. First, we generated a specific anti-Pcnt antiserum, which should detect all to date known Pcnt splice variants (Fig. 1, Fig. S3). Using the MmPeriC1 antiserum, we confirmed the previously reported localization of Pcnt at the BBCs in the dendritic knobs of the olfactory receptor neurons [15] (Fig. 3). For the retina, we described for the first time the presence of Pcnt at the BBC of the connecting cilium in the photoreceptors and at the centrioles of centrosomes of non-photoreceptor cells (Fig. 4, Fig. S1). To study the localization of Pcnt at the photoreceptor connecting cilium with high spatial resolution, we performed preembedding immunoelectron microscopy [24]. Photoreceptors are post-mitotic sensory neurons, which display a unique polarized structure. They are divided into a biosynthetically active inner segment and a photosensitive outer segment. The two segments are connected via a specialized bridge-like structure, the connecting cilium, which represents the transition zone of a prototypical primary cilium [19], [41]. At the ultrastructural level, Pcnt was present at the BB and the centriole of the BBC, where Pcnt is attached to the outer face of the microtubule doublets, and it was absent from the connecting cilium (Fig. 5). The cilium with its BBC is the only intracellular passage between the inner and outer segment of the photoreceptors. All molecules have to traffic from the synthesizing organelles of the nuclear and inner segment region through the connecting cilium to their destination in the outer segment. The import/export of molecules is thought to be regulated by proteins arranged in networks at the BBC. These protein networks handle the handover of cargos from the minus-end-directed microtubule-based transport through the inner segment to the molecular translocation machinery within the cilium [19], [42]. The localization of the multifunctional scaffold protein Pcnt at the strategic position of the BBC suggests its putative involvement in the regulation of this complex transport network.

To get a better understanding of the possible regulatory functions of Pcnt at the BBC, we examined in further immunocytochemical experiments the localization of known centrosomal and ciliary interaction partners of Pcnt at the connecting cilium (Fig. 6). CG-NAP and PCM1, two centrosomal interaction partners of Pcnt, were found at the base of the connecting cilium, the region where Pcnt is also present (Fig. 6A–H). At the centrosomes, CG-NAP interacts with Pcnt and both interact with the γ-TuRC (gamma tubulin ring complex). This complex forms a matrix, which is suggested to provide anchoring sites for various proteins, like e. g. for LIC (cytoplasmic dynein light intermediate chain) [43], and it serves as a targeting machinery for signaling enzymes like PKC and PKA [27], [44]. Another centrosomal protein, which directly interacts with Pcnt and is suggested to be involved in the microtubule- and dynactin-dependent recruitment of proteins to centrosomes [25], [26] and in primary cilia formation in cell culture [38], [39], is PCM1. In its interaction with PCM1 and CG-NAP and as a component of the protein complex associated with the γ-TuRC, Pcnt stabilizes microtubules and/or aids microtubule nucleation [26], [45]. Taken together, the known functions of Pcnt at the centrosome could be easily transferred to ciliary structures like the connecting cilium of photoreceptors in the retina [2], [44], [45].

Another interesting Pcnt interaction partner, which we identified for the first time at the BBC of photoreceptors, is DISC1 (Fig. 6I). The interaction between DISC1 and Pcnt at the centrosome in mammalian cells is known [31], and recent studies showed that DISC1 is involved in regulating the formation and/or maintenance of primary cilia and in establishing the subtype-specific targeting of dopamine receptors to the ciliary surface [34]. Questions, which we want to follow up in future studies, are whether DISC1 and Pcnt interact at the BBC of the photoreceptor and whether they are also involved in the targeting of 7-transmembrane domain receptors to the outer segment of photoreceptors.

Two known interaction partners of Pcnt at primary cilia are IFT20 and PC2 [12]. Recently, IFT20 has been described at the BBC of the photoreceptor connecting cilium [46], and in this study, we found PC2, a Ca^2+^ channel produced by the polycystic kidney disease 2 (*PKD2*) gene, at the connecting cilium of photoreceptors. In kidney cells, normally PC2 forms together with PC1 a cilium-located channel complex that raises intracellular Ca^2+^ in response to cilia bending [47], but also PC1-independent functions of PC2 in primary cilia were discussed [48]. At the connecting cilium, Ca_v_1.3 (α_1D_) is the only known Ca^2+^ channel subunit to date [49]. PC2, therefore, may be a potential candidate for the regulation of Ca^2+^- dependent processes at the connecting cilium of photoreceptors, e.g. the binding of Ca^2+^-binding proteins like centrins to the G-protein transducin [36], [50], [51].

Endoh-Yamagami and co-workers (2010) isolated in a forward genetic screen a frame shift mutation in a mouse line (line 239) by which all three pericentrin isoforms are disrupted. Homozygous mutant mice display a progressive embryonic growth retardation and perinatal lethality. Thus the mouse line 239 is suitable to investigate ciliogenesis in olfactory sensory neurons, which develop between E11 to E14 [52], but not to study ciliogenesis in rod photoreceptors, which happens postnatally. In a vital mouse line with a hypomorphic Pcnt mutation, a disturbed cilia assembly in the receptor neurons of the olfactory epithelium but not in the photoreceptors of the retina was reported [15]. What might be the reason for this difference in the two ciliated sensory tissues? The answer may be the specific expression of different Pcnt splice transcripts in the two ciliary tissues. RT-PCR analysis revealed that Pcnt B is expressed in all tested tissues (Fig. 7A and 8E), but shows a relatively low expression in photoreceptors (Fig. 8E). Expression of Pcnt S was found in the retina and in NIH 3T3 mouse fibroblasts, but not in the olfactory epithelium (Fig. 7A). Pcnt S is strongly expressed in photoreceptors (Fig. 8E). In Western blot analyses, we detected in the olfactory epithelium, the retina, and the NIH 3T3 mouse fibroblasts two protein bands corresponding to the Pcnt splice variants (Fig. 7B). In all three extracts a high molecular weight band of approximately 360 kDa corresponding to the holo protein Pcnt B was present (Fig. 1A). However, the second lower molecular weight protein band differed between the tissues. Whereas in the olfactory epithelium and the NIH 3T3 mouse fibroblasts extracts a 240–250 kDa protein band was present, representing the Pcnt splice variants A and/or S, in the retina extract a protein band with a molecular weight of about 225 kDa was found (Fig. 7B). Based on our results we suggest that this Pcnt form is a modified variant of Pcnt S. Separation of the photoreceptor layer from the rest of the retina by laser microdissection and subsequent RT-PCR and Western blot analysis demonstrated that photoreceptors preferentially express this smaller 225 kDa Pcnt variant (Fig. 8E–F). Taken together, these results suggest that in the olfactory epithelium and the retina different Pcnt splice variants execute their function, a possible explanation why ciliary assembly in photoreceptors is not affected by the decreased expression of the 360 kDa Pcnt B splice variant [15]. The patchwork of different Pcnt splice transcripts, individually expressed in distinct tissues or specialized parts of tissues, seems to reflect the complexity of Pcnt function, an assumption corroborated by the heterogeneous clinical manifestations associated with mutations in the Pcnt gene [10].

## Materials and Methods

### Ethics Statement

The experiments were performed in compliance with the guidelines for the welfare of experimental animals issued by the Federal Government of Germany, and the University of Erlangen-Nuremberg. The animal experiments were approved and registered by the Amt für Veterinärwesen der Stadt Erlangen (AZ: TS - 10/07 Lehrstuhl für Zoologie- Tierphysiologie). Mouse breeding was performed in the animal facilities of academy of natural Science and Medicine University of Erlangen-Nuremberg according to European and German (Tierschutzgesetz) guidelines for the welfare of experimental animals (AZ 820-8791.2.63).

### Animals

Adult C57BL/6 mice (age 2–4 months) maintained on a 12/12-h light/dark cycle with light on at 6 a.m. and with food and water ad libitum were used for these studies.

### Tissue preparation

Mice were deeply anesthetized with Isofluran and decapitated. The eyes were taken out. For preparation of the olfactory epithelium the skull was cleaved lengthwise and the epithelium was taken out of the nasal cavity on both sides of the skull.

For photoreceptor segregation, the retina was isolated and rubbed with the photoreceptor side on poly-D-lysine coated coverslips. Detached photoreceptors on the coverslips were immediately fixed for 10 minutes with pure ice cold EGTA saturated methanol at −20°C for the following immunocytochemical stainings.

For light microscopy, the eyes/the olfactory epithelia were mounted in a cup filled with freezing medium (Reichert-Jung, Bensheim, Germany) and frozen immediately in pure Isopentane cooled in liquid nitrogen for 45 seconds. Afterwards the frozen cups with the eyes/the olfactory epithelia are transferred directly into liquid nitrogen for a few minutes. Afterwards the frozen cups can be stored by −80°C until sectioning. The eyes/the olfactory epithelia were sectioned vertically with a cryostat at a thickness of 12 µm. The sections were collected on superfrost plus slides (Menzel Gläser, Braunschweig, Germany) for the following immunocytochemical stainings.

For preembedding immunoelectron microscopy, the eyes were taken out and put into a dish with ice cold phosphate-buffered saline (PBS; 0,01 M, pH 7.4). The eyes were opened along the ora serrata, and the cornea, lens and vitreous body were removed. The remaining eyecups with the attached retinae were fixed for 50 minutes in 4% paraformaldehyde (PFA) in PBS at room temperature (RT). Afterwards the eyecups were washed 3×15 minutes in PBS on ice and incubated in 10% sucrose in PBS for 1 hour on ice. Then the retinae were dissected free and further cryoprotected in 20% sucrose in PBS on ice for 1 hour and in 30% sucrose in PBS overnight at 4°C. Next day the retinae were cut in quarters and repeatedly frozen with liquid nitrogen and thawed to achieve a better penetration of the antibodies. After washing the retina in PBS on ice for 3×15 minutes, the retina quarters were embedded in 2% agar, and vertical sections (50–100 µm thick) were cut with a vibratome (Leica VT1000S, Wetzlar, Germany). The sections were collected in cold PBS for the following immunocytochemical stainings.

### Laser capture microdissection

For laser capture microdissection, eyes were prepared, frozen and sectioned at a thickness of 30 µm like described above for light microscopy. The serial sections were collected on membrane covered slides (MembraneSlide 1.0 PEN, Carl Zeiss MicroImaging, Munich, Germany). After sections were dry, they were fixed for 2 minutes in −20°C cooled 70% ethanol and washed in ultra-pure water at RT. Sections were stained in 1% cresyl violet in 50% ethanol for 20 seconds at RT and finally washed two times in 70% ethanol at RT. When sections were dry they were ready for microdissection and were dissected on the same day using the PALM micro Beam system (Carl Zeiss MicroImaging) equipped with a nitrogen laser (337 nm) for cutting and ejecting parts of the photoreceptor cell layer or of the residual retinal layers by laser pressure catapulting technology. After microdissection, the dissected samples were ejected from the object plane with a single laser shot and catapulted directly into a microtube cap (AdhesiveCap, Carl Zeiss MicroImaging) for subsequent sample preparation. Samples were collected from approximately 30 sections.

### Cell culture

NIH 3T3 cells (swiss mouse embryo fibroblast) were cultured in 10 cm dishes in Dulbecco's modified Eagle minimal essential medium (DMEM) supplemented with 10% fetal calf serum (FCS) (GibcoBRL, Invitrogen, Darmstadt) at 37°C in a humidified atmosphere of 5% CO_2_. Cells grown to about 80–90% confluency were rinsed once with PBS, incubated with 1 ml trypsin for 5 minutes at 37°C and passaged to new dishes with fresh DMEM/10% FCS.

For primary cilia formation cells grown to about 50–70% confluency in DMEM/10% FCS were washed once with PBS and harvested further 3–4 days in DMEM without FCS. Because of the lack of FCS most of the cells stop to divide and rest in the G_0_-phase, where primary cilia formation occurs.

For light microscopy, poly-D-lysine coated coverslips were placed in a 6 well plate and cells were harvested in the wells like described above. 80–90% confluent cells were washed once with PBS and fixed 10 minutes in methanol at −20°C. After rinsing for two times with PBS for 10 minutes, the coverslips with the adherent cells are transferred to a staining chamber for the following immunocytochemical stainings.

### RT-PCR

Fresh isolated tissue/microdissected tissue was homogenized in RLT buffer (Qiagen, Hilden, Germany) containing 1% β-Mercaptoethanol. RNA was isolated using the RNeasy Mini Kit (whole tissue) or the RNeasy Micro Kit (microdissected tissue) (Qiagen). Poly(dT)- and random hexamer-primed cDNA synthesis (reverse transcriptase reaction) was performed 1 hour at 40°C using 5x RT-buffer, a mixture of dNTPs, RNAsin (all from Promega, Mannheim, Germany) and 1 µg of total RNA (whole tissue) or the complete RNA (microdissected tissue). PCR was performed in a volume of 25 µl using 1 µl (whole tissue) or 3 µl (microdissected tissue) of prepared cDNA and 1 µl of each primer (of the working solution 10 pM)/reaction. Cycling conditions were 40 cycles at 95°C for 45 seconds, 55–63°C for 45 seconds, and 72°C for 1 minute followed by a 10 minute 72°C extension step. PCR product lengths were determined on 1% agarose gels. As DNA marker, a 1-kb DNA ladder (Fermentas, St. Leon-Rot, Germany) was used. Sequencing of PCR products was performed by GATC (Konstanz, Germany). For sequence comparisons and oligonucleotide generation the computer program OmigaTM Version 2.0 (Oxford Molecular Ltd., Oxford, UK) was used. Primers specific for mouse Pcnt splice transcripts were as follows (used for RT-PCR and DNA sequencing): Primer set specific for Pcnt S (250) with forward primer (5′-CTGGCTATTGACCCTGAT-3′) and reverse primer (5′-GCTCTGGTAGACTCTCCTA-3′), primer set specific for Pcnt B (360) with forward primer (5′-TCCGTAAGAGATAGCCTGAG-3′) and reverse primer (5′-GCTCTGGTAGACTCTCCTA-3′). Primers specific for β-Actin were as follows (used for RT-PCR): Primer set specific for β-Actin with forward primer (5′-TCACCCACACTGTGCCCATCTACGAG-3′) and reverse primer (5′-ACACAGAGTACTTGCGCTCAGGAGGA-3′).

### Western blot

Fresh isolated tissues/microdissected photoreceptors/NIH 3T3 cells were homogenized in extraction lysis buffer (50 mM Tris-HCl (pH 7.5), 150 mM NaCl, 1% Triton X-100, 0.5% Natrium-Deoxycholat). Proteins were separated by SDS-PAGE using NuPAGE Novex Tris-Acetate Mini Gels (Invitrogen, Darmstadt), transferred electrophoretically by 0.2 A at 4°C over night in a tank blot chamber (Trans-Blot Cell, Bio-Rad Laboratories, USA) to polyvinylidene difluoride (PVDF) membranes (Amersham Hybond-P, GE Healthcare, Munich, Germany) and probed with primary and horse radish peroxidase (HRP) labeled secondary antibodies (Sigma-Aldrich, St. Louis, MO). Pictures were obtained with a molecular imager (ChemiDoc XRS, Bio-Rad Laboratories).

### Immunofluorescence microscopy

Sections/isolated photoreceptors/NIH 3T3 cells were incubated with 0.01% Tween 20 in PBS for 10 minutes and washed 10 minutes in PBS. Afterwards samples were blocked for 45 minutes in blocking solution (0.5% cold-water fish gelatin and 0.1% ovalbumin in PBS), followed by overnight incubation with primary antibodies diluted in blocking solution at 4°C. Next day the 3×10 minutes in PBS washed samples were incubated with secondary antibodies Alexa^TM^ 594 (red fluorescence) and Alexa^TM^ 488 (green fluorescence) goat anti-mouse, goat anti-rabbit IgG (H+L) conjugates (1∶500; Molecular Probes, Eugene, OR) in blocking solution with DAPI (4,6-diamidino-2-phenylindole) (1∶50000, Sigma-Aldrich, St. Louis, MO). After further 3×10 minutes PBS washes, samples were mounted in Aqua Poly Mount (Polysciences, Eppelheim, Germany) and analyzed with a Zeiss Axio Imager Z1 equipped with an ApoTome (Zeiss, Oberkochen, Germany). Projections of picture stacks were calculated with AxioVision 4.8 software (Zeiss). The images were adjusted for contrast and brightness using Adobe Photoshop CS (Adobe, San Jose, CA). We performed three-dimensional reconstruction of cilia using Imaris software (Bitplane, Zurich) with picture stacks from AxioVision 4.8 software (Zeiss).

### Immunoelectron microscopy

Vibratome sections were blocked in 10% normal goat serum and 1% bovine serum albumin in PBS for 2 hours. Sections were incubated with the primary antibody against Pcnt for 4 days at 4°C. After 4×15 minutes PBS washes, binding sites of the primary antibody were visualized with a biotinylated goat anti-rabbit IgG secondary antiserum (Vector Laboratories, Burlinggame, CA) diluted 1∶100 and a peroxidase-based enzymatic detection system (Vectastain Elite ABC kit; Vector Laboratories). The provided avidin-biotin HRP complex (ABC) binds the biotinylated secondary antibodies. This immunocomplex was visualized by adding 0.01% hydrogen peroxide to a 0.05% 3.3'-diaminobenzidine (DAB) solution. The staining was fixed by incubation in 2.5% glutaraldehyde in cacodylate buffer (0.1 M, pH 7.4) for 1 hour. DAB precipitates were silver intensified and sections were finally incubated in 0.5% OsO_4_ in cacodylate buffer for 30 minutes at 4°C. After washing and dehydration vibratome sections were flat-mounted between two sheaths of heat resistant transparency film in Epon. Ultrathin sections were examined and photographed with a Zeiss EM10 electron microscope (Zeiss) and a Gatan SC1000 OriusTM CCD camera (GATAN, Munich, Germany) in combination with the DigitalMicrographTM software (GATAN, Pleasanton, CA). Images were adjusted for contrast and brightness using Adobe Photoshop CS (Adobe).

### Generation of Pcnt specific antibodies

An 843 bp Pcnt cDNA part was cloned from reverse transcription (RT)-PCR products into the pGEX-4T3 expression vector (GE Healthcare) using BamHI and XhoI restriction sites. Expression and purification of the GST fusion protein was performed according to the manufacturer's instructions (GE Healthcare). After cleavage of the fusion protein with thrombin on the column Pcnt was eluted with PBS. Peptide immunization of rabbits was accomplished by a company (Pineda Antikörper-Service, Berlin, Germany). Polyclonal antisera (MmPeriC1) from rabbit against recombinantly expressed mouse Pcnt were affinity-purified on high trap N-hydroxysuccinimide columns (GE Healthcare).

### Antibodies

For light and preembedding immunoelectron microscopy and for Western blotting the following antibodies were used: rabbit anti-Pcnt MmPeriC1 pAb (light microscopy 1∶500, preembedding immunoelectron microscopy 1∶400, Western blot 1∶250); rabbit anti-Pcnt pAb (light microscopy 1∶100, Western blot 1∶1000, Cat. No. PRB-432C); mouse anti-ac. tubulin mAb clone 6-11B-1 (light microscopy 1∶600, Cat. No. T6793), mouse anti-Opsin mAb clone RET-P1 (light microscopy 1∶1000, Cat. No. O4886), mouse anti-α-tubulin mAb clone DM1A (light microscopy 1∶700, Cat. No. T9026), mouse anti-γ-tubulin mAb clone GTU-88 (light microscopy 1∶200, Cat. No. T6557), rabbit anti-IFT20 (light microscopy 1∶100, Cat. No. HPA021376) (all Sigma-Aldrich, St. Louis, MO), rabbit anti-DISC1 (light microscopy 1∶100, Cat. No. AHP1446, AbD Serotec, Düsseldorf, Germany), mouse anti-centrin clone 20H5 (light microscopy 1∶100, Cat. No. 04-1624) and rabbit anti-PC2 (light microscopy 1∶100, Cat. No. AB9088) (both Millipore GmbH Schwalbach/Ts, Germany), mouse anti-centrin Cen3 mAb (light microscopy 1∶2) was used as a molecular marker for the ciliary apparatus of photoreceptors [53], rabbit anti-CG-NAP (light microscopy 1∶1000) was a gift from Mikiko Takahashi and Yoshitaka Ono (Biosignal Research Center, Kobe University, Japan) previously described in [2], rabbit anti-PCM1 (light microscopy 1∶600) was a gift from Andreas Merdes (CNRS-Pierre Fabre, Toulouse, France) previously described in [26].

## Supporting Information

Figure S1 **3 D reconstruction of Pericentrin at connecting cilia and at centrosomes in the mouse retina.** (A–C) High magnification micrographs of a single connecting cilium (CC) in a cryostat section through an adult mouse retina double labeled with the MmPeriC1 antiserum against Pcnt (A and C, green) and an antibody against Cen3 (B and C, red). Pcnt colocalizes partially with Cen 3 at the BBC of the CC. (D–E) High resolution 3D reconstruction demonstrates that Pcnt (green) ensheaths the BBC of the photoreceptor CC (D, red, Cen 3) and the centrioles (C) of the centrosomes of non-photoreceptor cells (E, red, Cen 3). (F–H) High magnification micrographs of a single connecting cilium (CC) in a cryostat section through an adult mouse retina double labeled with the MmPeriC1 antiserum against Pcnt (F and H, green) and an antibody against ac. tubulin (G and H, red). Pcnt colocalizes partially with ac. tubulin at the BBC of the CC. (I) High resolution 3D reconstruction demonstrates that Pcnt (green) ensheaths the BBC of the CC (red, ac. tubulin). Scale bars: 1 µm (C and H).
(TIF)

Figure S2 **Localization of Pericentrin in mouse photoreceptors.** (A) Differential interference micrograph of an isolated mouse photoreceptor. (B–D) An isolated photoreceptor double-labeled with the MmPeriC1 antiserum (Pcnt, B, green) and Opsin as a marker for the photoreceptor outer segment (OS) (C, red). (D) The merge of the stainings and the additional labeling of the photoreceptor nucleus with DAPI demonstrate the localization of Pcnt at the region of the basal body complex (BBC) at the apical site of the photoreceptor inner segment (IS). Scale bar: 5 µm (A).
(TIF)

Figure S3 **Detection of Pericentrin splice variants with different antibodies.** (A) The MmPeriC1 antiserum shows the recombinantly expressed Pcnt peptide used for immunization with the molecular weight of ∼36 kDa. (B) Western blot analysis with the MmPeriC1 antiserum of protein extracts of retina, olfactory epithelium, and E14.5 brain. In the retina two major bands are recognized at 360 kDa and 225 kDa (black arrowhead). In the olfactory epithelium and the E14.5 brain extract up to 3 bands (about 360 kDa and 250 kDa, black arrows) could be detected. (C) Western blot analysis using the polyclonal Pcnt pAb (Covance), which was raised against an epitope common to all three isoforms. This antiserum detects the same Pcnt bands as shown in (B). Additionally, the antiserum shows in all extracts a protein band with a molecular weight above 500 kDa (grey arrow).
(TIF)

## References

[1] Doxsey SJ, Stein P, Evans L, Calarco PD, Kirschner M (1994). Pericentrin, a highly conserved centrosome protein involved in microtubule organization.. Cell.

[2] Takahashi M, Yamagiwa A, Nishimura T, Mukai H, Ono Y (2002). Centrosomal proteins CG-NAP and kendrin provide microtubule nucleation sites by anchoring gamma-tubulin ring complex.. Mol Biol Cell.

[3] Zimmerman WC, Sillibourne J, Rosa J, Doxsey SJ (2004). Mitosis-specific anchoring of gamma tubulin complexes by pericentrin controls spindle organization and mitotic entry.. Mol Biol Cell.

[4] Flory MR, Davis TN (2003). The centrosomal proteins pericentrin and kendrin are encoded by alternatively spliced products of one gene.. Genomics.

[5] Miyoshi K, Asanuma M, Miyazaki I, Matsuzaki S, Tohyama M (2006). Characterization of pericentrin isoforms in vivo.. Biochem Biophys Res Commun.

[6] Piane M, Della Monica M, Piatelli G, Lulli P, Lonardo F (2009). Majewski osteodysplastic primordial dwarfism type II (MOPD II) syndrome previously diagnosed as Seckel syndrome: report of a novel mutation of the PCNT gene.. Am J Med Genet A.

[7] Endoh-Yamagami S, Karkar KM, May SR, Cobos I, Thwin MT (2010). A mutation in the pericentrin gene causes abnormal interneuron migration to the olfactory bulb in mice.. Dev Biol.

[8] Gillingham AK, Munro S (2000). The PACT domain, a conserved centrosomal targeting motif in the coiled-coil proteins AKAP450 and pericentrin.. EMBO Rep.

[9] Carafoli E, Santella L, Branca D, Brini M (2001). Generation, control, and processing of cellular calcium signals.. Crit RevBiochemMolBiol.

[10] Delaval B, Doxsey SJ (2010). Pericentrin in cellular function and disease.. J Cell Biol.

[11] Satir P, Pedersen LB, Christensen ST (2010). The primary cilium at a glance.. J Cell Sci.

[12] Jurczyk A, Gromley A, Redick S, San Agustin J, Witman G (2004). Pericentrin forms a complex with intraflagellar transport proteins and polycystin-2 and is required for primary cilia assembly.. J Cell Biol.

[13] Miyoshi K, Onishi K, Asanuma MP, Miyazaki I, Diaz-Corrales FJ (2006). Embryonic expression of pericentrin suggests universal roles in ciliogenesis.. Dev Genes Evol.

[14] Martinez-Campos M, Basto R, Baker J, Kernan M, Raff JW (2004). The Drosophila pericentrin-like protein is essential for cilia/flagella function, but appears to be dispensable for mitosis.. J Cell Biol.

[15] Miyoshi K, Kasahara K, Miyazaki I, Shimizu S, Taniguchi M (2009). Pericentrin, a centrosomal protein related to microcephalic primordial dwarfism, is required for olfactory cilia assembly in mice.. FASEB J.

[16] Rauch A, Thiel CT, Schindler D, Wick U, Crow YJ (2008). Mutations in the pericentrin (PCNT) gene cause primordial dwarfism.. Science.

[17] Willems M, Genevieve D, Borck G, Baumann C, Baujat G (2010). Molecular analysis of pericentrin gene (PCNT) in a series of 24 Seckel/microcephalic osteodysplastic primordial dwarfism type II (MOPD II) families.. J Med Genet.

[18] Kennedy B, Malicki J (2009). What drives cell morphogenesis: a look inside the vertebrate photoreceptor.. Dev Dyn.

[19] Roepman R, Wolfrum U (2007). Protein networks and complexes in photoreceptor cilia.. Subcell Biochem.

[20] Pazour GJ, Baker SA, Deane JA, Cole DG, Dickert BL (2002). The intraflagellar transport protein, IFT88, is essential for vertebrate photoreceptor assembly and maintenance.. JCell Biol.

[21] Gießl A, Pulvermüller A, Trojan P, Park JH, Choe HW (2004). Differential expression and interaction with the visual G-protein transducin of centrin isoforms in mammalian photoreceptor cells.. JBiolChem.

[22] Insinna C, Pathak N, Perkins B, Drummond I, Besharse JC (2008). The homodimeric kinesin, Kif17, is essential for vertebrate photoreceptor sensory outer segment development.. Dev Biol.

[23] Reidel B, Goldmann T, Gießl A, Wolfrum U (2008). The translocation of signaling molecules in dark adapting mammalian rod photoreceptor cells is dependent on the cytoskeleton.. Cell Motil Cytoskeleton.

[24] Sedmak T, Sehn E, Wolfrum U (2009). Immunoelectron microscopy of vesicle transport to the primary cilium of photoreceptor cells.. Methods Cell Biol.

[25] Li Q, Hansen D, Killilea A, Joshi HC, Palazzo RE (2001). Kendrin/pericentrin-B, a centrosome protein with homology to pericentrin that complexes with PCM-1.. J Cell Sci.

[26] Dammermann A, Merdes A (2002). Assembly of centrosomal proteins and microtubule organization depends on PCM-1.. JCell Biol.

[27] Chen D, Purohit A, Halilovic E, Doxsey SJ, Newton AC (2004). Centrosomal anchoring of protein kinase C betaII by pericentrin controls microtubule organization, spindle function, and cytokinesis.. J Biol Chem.

[28] Doxsey S, Zimmerman W, Mikule K (2005). Centrosome control of the cell cycle.. Trends in Cell Biology.

[29] Kawaguchi S, Zheng Y (2004). Characterization of a Drosophila centrosome protein CP309 that shares homology with Kendrin and CG-NAP.. Mol Biol Cell.

[30] Balczon R, Bao L, Zimmer WE (1994). PCM-1, A 228-kD centrosome autoantigen with a distinct cell cycle distribution.. J Cell Biol.

[31] Miyoshi K, Asanuma M, Miyazaki I, Diaz-Corrales FJ, Katayama T (2004). DISC1 localizes to the centrosome by binding to kendrin.. Biochem Biophys Res Commun.

[32] Shimizu S, Matsuzaki S, Hattori T, Kumamoto N, Miyoshi K (2008). DISC1-kendrin interaction is involved in centrosomal microtubule network formation.. Biochem Biophys Res Commun.

[33] Li C, Inglis PN, Leitch CC, Efimenko E, Zaghloul NA (2008). An essential role for DYF-11/MIP-T3 in assembling functional intraflagellar transport complexes.. PLoS Genet.

[34] Marley A, von Zastrow M (2010). DISC1 regulates primary cilia that display specific dopamine receptors.. PLoS One.

[35] Goetz SC, Anderson KV (2010). The primary cilium: a signalling centre during vertebrate development.. Nat Rev Genet.

[36] Gießl A, Trojan P, Pulvermüller A, Wolfrum U (2004). Centrins, potential regulators of transducin translokation in photoreceptor cells.. Williams DS (ed) Cell biology and related disease of the outer retina World Scientific Publishing Company Pte Ltd.

[37] Shu X, Fry AM, Tulloch B, Manson FD, Crabb JW (2005). RPGR ORF15 isoform co-localizes with RPGRIP1 at centrioles and basal bodies and interacts with nucleophosmin.. HumMolGenet.

[38] Graser S, Stierhof YD, Lavoie SB, Gassner OS, Lamla S (2007). Cep164, a novel centriole appendage protein required for primary cilium formation.. J Cell Biol.

[39] Mikule K, Delaval B, Kaldis P, Jurcyzk A, Hergert P (2007). Loss of centrosome integrity induces p38-p53-p21-dependent G1-S arrest.. Nat Cell Biol.

[40] Chakarova CF, Khanna H, Shah AZ, Patil SB, Sedmak T (2011). TOPORS, implicated in retinal degeneration, is a cilia-centrosomal protein.. Hum Mol Genet.

[41] Gießl A, Regus-Leidig H, Brandstätter JH (2010). Signal transduction and signal transmission: The two faces of a photoreceptor.. e-Neuroforum.

[42] Sung CH, Tai AW (2000). Rhodopsin trafficking and its role in retinal dystrophies.. IntRevCytol.

[43] Purohit A, Tynan SH, Vallee R, Doxsey SJ (1999). Direct interaction of pericentrin with cytoplasmic dynein light intermediate chain contributes to mitotic spindle organization.. J Cell Biol.

[44] Diviani D, Langeberg LK, Doxsey SJ, Scott JD (2000). Pericentrin anchors protein kinase A at the centrosome through a newly identified RII-binding domain.. Curr Biol.

[45] Dictenberg JB, Zimmerman W, Sparks CA, Young A, Vidair C (1998). Pericentrin and gamma-tubulin form a protein complex and are organized into a novel lattice at the centrosome.. J Cell Biol.

[46] Sedmak T, Wolfrum U (2010). Intraflagellar transport molecules in ciliary and nonciliary cells of the retina.. J Cell Biol.

[47] Nauli SM, Alenghat FJ, Luo Y, Williams E, Vassilev P (2003). Polycystins 1 and 2 mediate mechanosensation in the primary cilium of kidney cells.. Nat Genet.

[48] Karcher C, Fischer A, Schweickert A, Bitzer E, Horie S (2005). Lack of a laterality phenotype in Pkd1 knock-out embryos correlates with absence of polycystin-1 in nodal cilia.. Differentiation.

[49] Kersten FF, van Wijk E, van Reeuwijk J, van der Zwaag B, Marker T (2010). Association of whirlin with Cav1.3 (alpha1D) channels in photoreceptors, defining a novel member of the usher protein network.. Invest Ophthalmol Vis Sci.

[50] Gießl A, Trojan P, Rausch S, Pulvermüller A, Wolfrum U (2006). Centrins, gatekeepers for the light-dependent translocation of transducin through the cell connecting cilium.. Vision Res.

[51] Hurd T, Zhou W, Jenkins P, Liu CJ, Swaroop A (2010). The retinitis pigmentosa protein RP2 interacts with polycystin 2 and regulates cilia-mediated vertebrate development.. Hum Mol Genet.

[52] Jenkins PM, McEwen DP, Martens JR (2009). Olfactory cilia: linking sensory cilia function and human disease.. Chem Senses.

[53] Evans RJ, Schwarz N, Nagel-Wolfrum K, Wolfrum U, Hardcastle AJ (2010). The retinitis pigmentosa protein RP2 links pericentriolar vesicle transport between the Golgi and the primary cilium.. Hum Mol Genet.

