# Supplementary figures and images for: The Centrosomal Protein Pericentrin Identified at the Basal Body Complex of the Connecting Cilium in Mouse Photoreceptors
Source: PLoS One. 2011 Oct 21;6(10):e26496. doi: 10.1371/journal.pone.0026496 (PMC3198765; doi:10.1371/journal.pone.0026496)

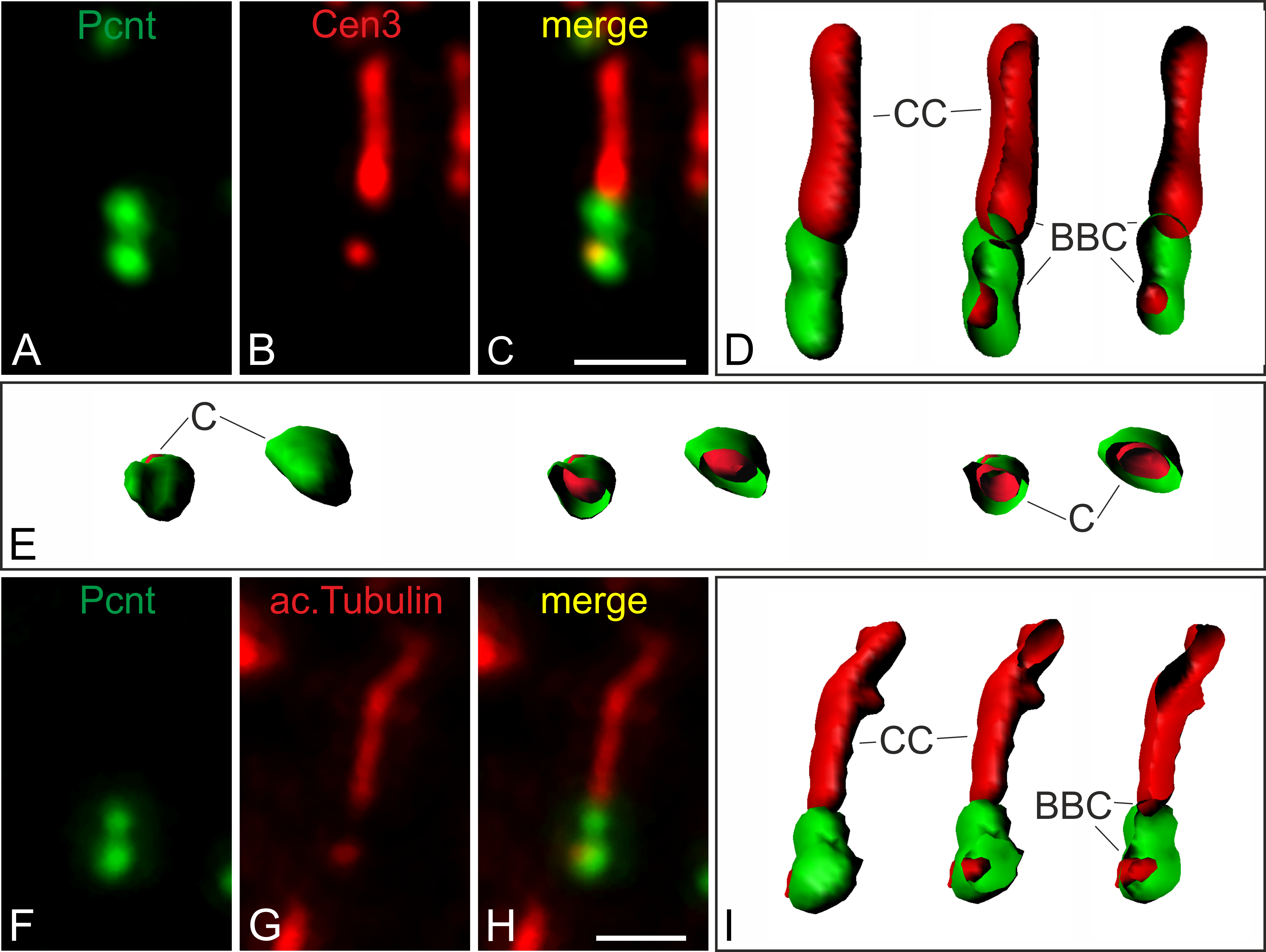

Supplement: Figure S1 — 3 D reconstruction of Pericentrin at connecting cilia and at centrosomes in the mouse retina. (A–C) High magnification micrographs of a single connecting cilium (CC) in a cryostat section through an adult mouse retina double labeled with the MmPeriC1 antiserum against Pcnt (A and C, green) and an antibody against Cen3 (B and C, red). Pcnt colocalizes partially with Cen 3 at the BBC of the CC. (D–E) High resolution 3D reconstruction demonstrates that Pcnt (green) ensheaths the BBC of the photoreceptor CC (D, red, Cen 3) and the centrioles (C) of the centrosomes of non-photoreceptor cells (E, red, Cen 3). (F–H) High magnification micrographs of a single connecting cilium (CC) in a cryostat section through an adult mouse retina double labeled with the MmPeriC1 antiserum against Pcnt (F and H, green) and an antibody against ac. tubulin (G and H, red). Pcnt colocalizes partially with ac. tubulin at the BBC of the CC. (I) High resolution 3D reconstruction demonstrates that Pcnt (green) ensheaths the BBC of the CC (red, ac. tubulin). Scale bars: 1 µm (C and H). (TIF) [file pone.0026496.s001.tif]

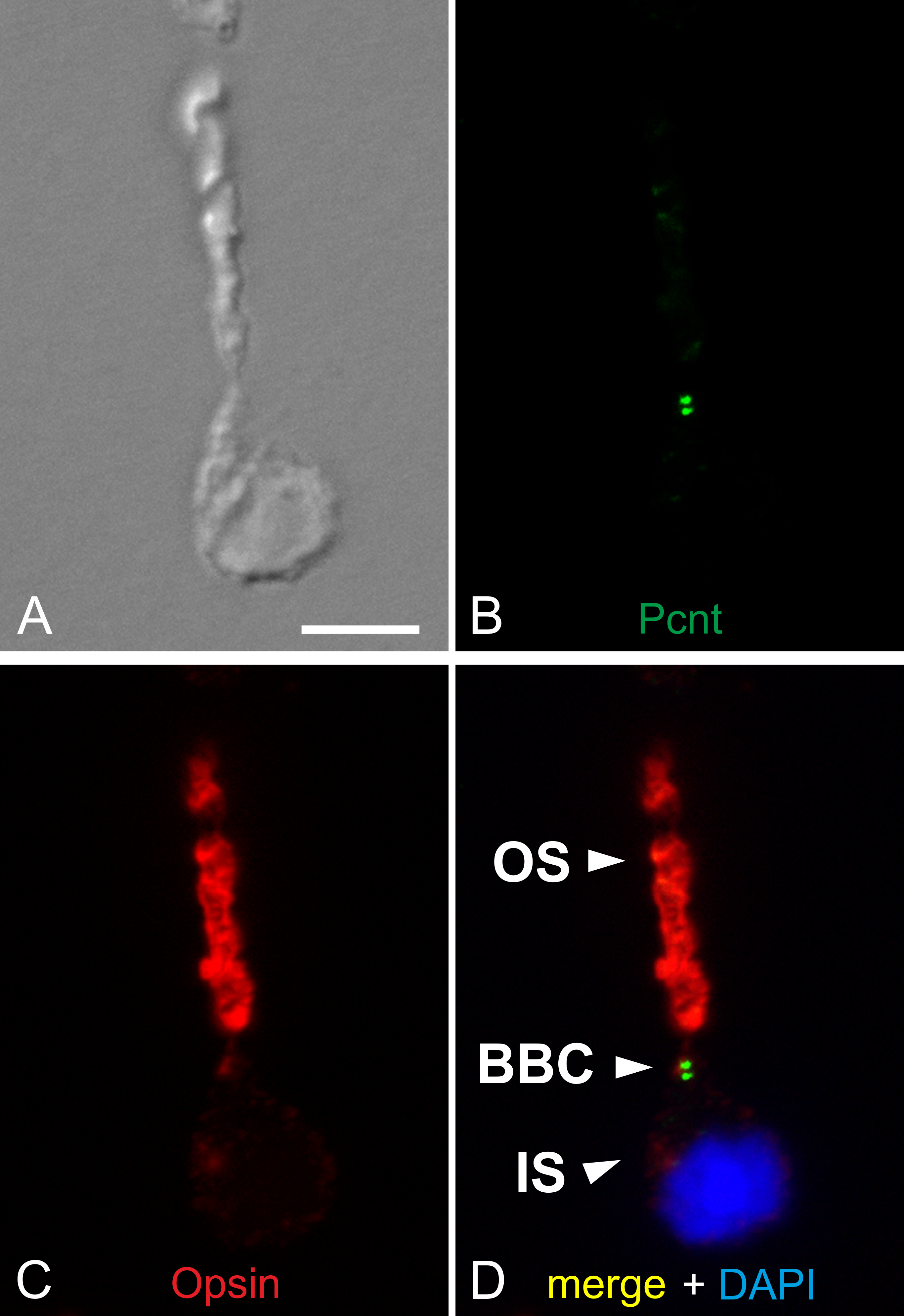

Supplement: Figure S2 — Localization of Pericentrin in mouse photoreceptors. (A) Differential interference micrograph of an isolated mouse photoreceptor. (B–D) An isolated photoreceptor double-labeled with the MmPeriC1 antiserum (Pcnt, B, green) and Opsin as a marker for the photoreceptor outer segment (OS) (C, red). (D) The merge of the stainings and the additional labeling of the photoreceptor nucleus with DAPI demonstrate the localization of Pcnt at the region of the basal body complex (BBC) at the apical site of the photoreceptor inner segment (IS). Scale bar: 5 µm (A). (TIF) [file pone.0026496.s002.tif]

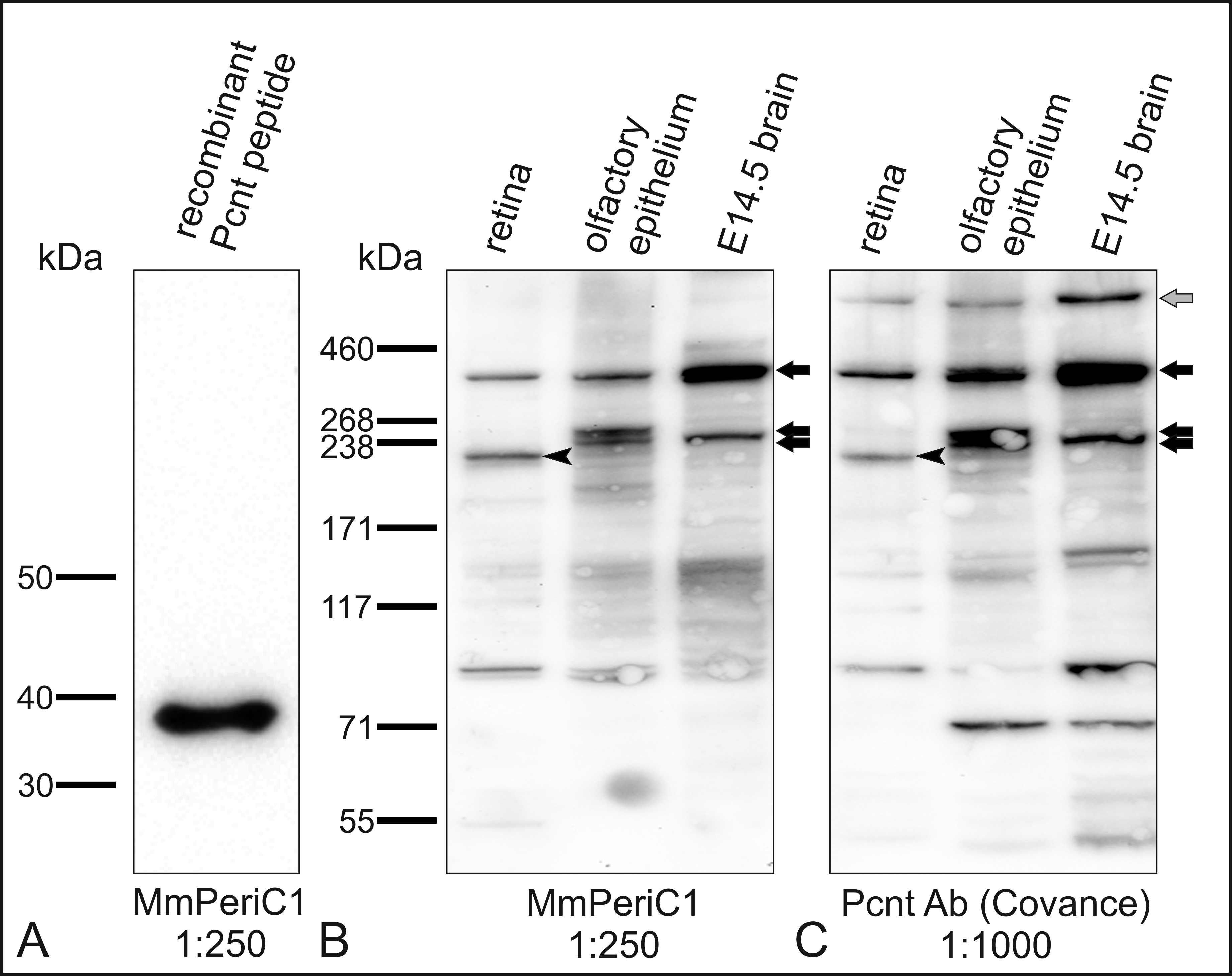

Supplement: Figure S3 — Detection of Pericentrin splice variants with different antibodies. (A) The MmPeriC1 antiserum shows the recombinantly expressed Pcnt peptide used for immunization with the molecular weight of ∼36 kDa. (B) Western blot analysis with the MmPeriC1 antiserum of protein extracts of retina, olfactory epithelium, and E14.5 brain. In the retina two major bands are recognized at 360 kDa and 225 kDa (black arrowhead). In the olfactory epithelium and the E14.5 brain extract up to 3 bands (about 360 kDa and 250 kDa, black arrows) could be detected. (C) Western blot analysis using the polyclonal Pcnt pAb (Covance), which was raised against an epitope common to all three isoforms. This antiserum detects the same Pcnt bands as shown in (B). Additionally, the antiserum shows in all extracts a protein band with a molecular weight above 500 kDa (grey arrow). (TIF) [file pone.0026496.s003.tif]
